# Supplementary figures and images for: Using Electrical Muscle Stimulation to Enhance Electrophysiological Performance of Agonist–Antagonist Myoneural Interface
Source: Bioengineering (Basel). 2024 Sep 10;11(9):904. doi: 10.3390/bioengineering11090904 (PMC11444137; doi:10.3390/bioengineering11090904)

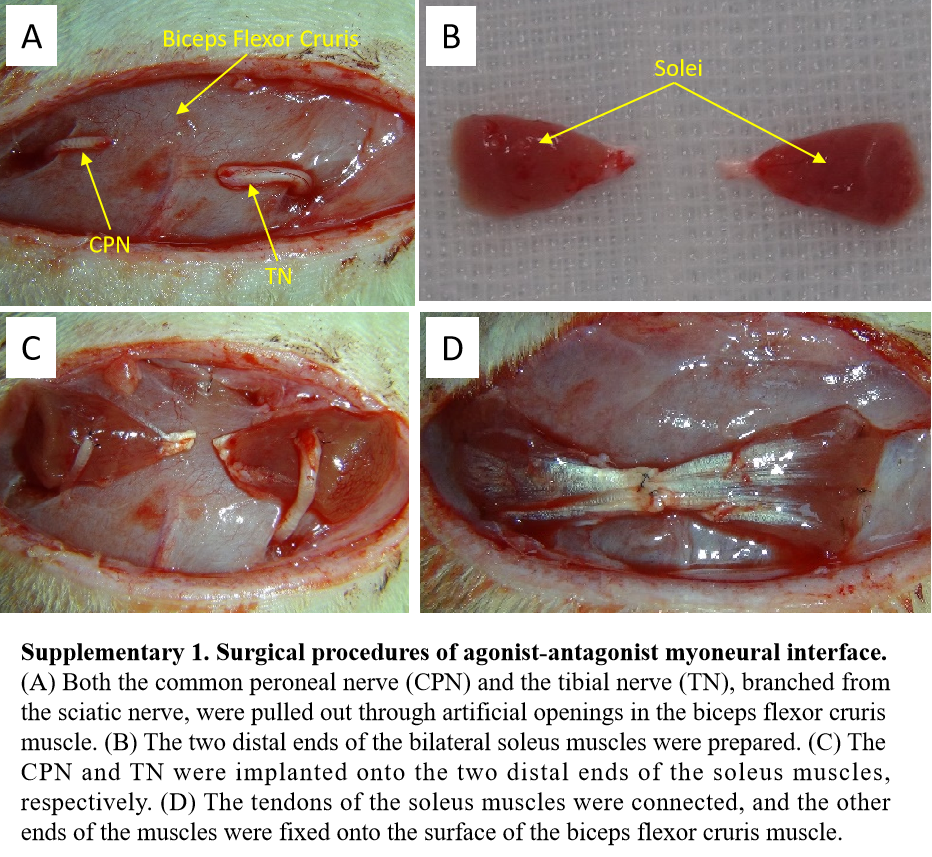

Supplement: Supplementary file 1 [file bioengineering-11-00904-s001.zip › Suplementary Figure S1 Main procedures of AMI surgery.png]
